# Mammalian Nudix proteins cleave nucleotide metabolite caps on RNAs

**DOI:** 10.1093/nar/gkaa402

**Published:** 2020-05-20

**Authors:** Sunny Sharma, Ewa Grudzien-Nogalska, Keith Hamilton, Xinfu Jiao, Jun Yang, Liang Tong, Megerditch Kiledjian

**Affiliations:** Department of Cell Biology and Neuroscience, Rutgers University, Piscataway, NJ 08854, USA; Department of Cell Biology and Neuroscience, Rutgers University, Piscataway, NJ 08854, USA; Department Biological Sciences, Columbia University, New York, NY 10027, USA; Department of Cell Biology and Neuroscience, Rutgers University, Piscataway, NJ 08854, USA; Department of Cell Biology and Neuroscience, Rutgers University, Piscataway, NJ 08854, USA; Department Biological Sciences, Columbia University, New York, NY 10027, USA; Department of Cell Biology and Neuroscience, Rutgers University, Piscataway, NJ 08854, USA

## Abstract

We recently reported the presence of nicotinamide adenine dinucleotide (NAD)-capped RNAs in mammalian cells and a role for DXO and the Nudix hydrolase Nudt12 in decapping NAD-capped RNAs (deNADding) in cells. Analysis of 5′caps has revealed that in addition to NAD, mammalian RNAs also contain other metabolite caps including flavin adenine dinucleotide (FAD) and dephosphoCoA (dpCoA). In the present study we systematically screened all mammalian Nudix proteins for their potential deNADing, FAD cap decapping (deFADding) and dpCoA cap decapping (deCoAping) activity. We demonstrate that Nudt16 is a novel deNADding enzyme in mammalian cells. Additionally, we identified seven Nudix proteins—Nudt2, Nudt7, Nudt8, Nudt12, Nudt15, Nudt16 and Nudt19, to possess deCoAping activity *in vitro*. Moreover, our screening revealed that both mammalian Nudt2 and Nudt16 hydrolyze FAD-capped RNAs *in vitro* with Nudt16 regulating levels of FAD-capped RNAs in cells. All decapping activities identified hydrolyze the metabolite cap substrate within the diphosphate linkage. Crystal structure of human Nudt16 in complex with FAD at 2.7 Å resolution provide molecular insights into the binding and metal-coordinated hydrolysis of FAD by Nudt16. In summary, our study identifies novel cellular deNADding and deFADding enzymes and establishes a foundation for the selective functionality of the Nudix decapping enzymes on non-canonical metabolite caps.

## INTRODUCTION

Cellular metabolism and gene expression are two fundamental biological processes that share an intricate relationship, coordination of which is essential for the survival of all living organisms (1). Apart from signal transduction pathways that can target key transcription factors, several key metabolites like nicotinamide adenine dinucleotide (NAD), acetyl coenzyme A (acetyl-CoA), S-adenosyl-methionine and flavin adenine dinucleotide (FAD) can directly regulate gene expression by serving as cofactors or co-substrates for many enzymes that modify chromatin and play key roles in activation and repression of gene expression (1).

The recent identification of 5′end NAD caps on mRNA in bacteria, yeast, plants and mammals has further expanded our understanding of the relationship between nucleotide metabolites and a potential direct role in gene expression (2–5). Studies in bacteria have shown that incorporation of an NAD cap at the 5′end appears to be during transcription initiation. Instead of canonical transcription initiation with adenosine triphosphate (ATP) as the first transcribed nucleotide, bacterial RNA polymerase can add NAD as a ‘non-canonical initiating nucleotide’ (6). Studies aimed at understanding the function of these non-canonical caps, both in bacteria and mammalian cells, have led to the identification of NAD-cap decapping (deNADding) enzymes—NudC in bacteria, its homolog Nudt12 and the non-canonical decapping enzyme, DXO in mammals (2–3,7–8). Bacteria harboring a deleted *NudC* gene accumulate NAD-capped RNAs (2) that appear to stabilize the RNA (6), suggesting the NAD cap my function analogous to the mammalian m^7^G cap. While NAD caps protect RNA from 5′end degradation in bacteria, in mammals NAD caps promote RNA decay (3). Similar to canonical decapping enzymes that each preferentially target a subset of mRNAs (9), Nudt12 and DXO deNAD distinct subsets of NAD-capped mRNAs. Nudt12 functions on a class of nuclear-encoded mitochondrial protein mRNAs containing an NAD-cap (8) and also possesses canonical m^7^G-cap decapping activity on circadian clock mRNAs (10). The most prevalent NAD-capped RNA substrates for DXO are a set of intronic NAD-capped small nucleolar (sno)RNAs that are elevated in the absence of DXO (3). Importantly, detection of NAD-capped intronic snoRNAs in cells devoid of DXO indicate that NAD caps can also be added to 5′ processed termini suggestive of an NAD capping mechanism in mammalian cells (3).

A systems-level LC-MS/MS based, CapQuant analysis of cap epitranscriptome from different organisms including mammalian cells has recently revealed that in addition to NAD, mRNAs also contain other metabolite caps including FAD, uridine diphosphate glucose (UDP-Glc), and uridine diphosphate *N*-acetylglucosamine (UDP-GlcNAc) (11). Nevertheless, the function of these metabolite caps in RNA metabolism and which enzymes can hydrolyze them remain unknown.

The Nucleoside diphosphate linked to another moiety, X (Nudix) superfamily of proteins constitute pyrophosphohydrolases of substrates that include (d)NTPs, nucleotide sugars, capped (m^7^Gppp and Gppp) RNAs and dinucleotide coenzymes including NAD, FAD, dephosphoCoA (dpCoA) and their derivatives (12). Functional and mutational analysis of these proteins have led to the identification of a highly conserved 23-amino acids Nudix motif (Nudix box), Gx_5_Ex_5_[UA]xREx_2_EExGU, where U is an aliphatic, hydrophobic residue (12). Two of the deNADding enzymes, Nudt12 and its bacterial homolog NudC, belong to the Nudix protein family (2,8,13).

In the present study, we examine all mammalian Nudix proteins for potential decapping activity on NAD-, dpCoA- and FAD-capped RNAs. We demonstrate that apart from Nudt12 and DXO, Nudt16 is a novel mammalian deNADding enzyme. Analysis of total NAD-capped RNA levels in a Nudt16 knockout cell line further validated the deNADding activity of Nudt16 in cells. Additionally, we identify seven Nudix proteins (Nudt2, Nudt7, Nudt8, Nudt15, Nudt16 and Nudt19) that possess dpCoA cap decapping (deCoAping) activity *in vitro*, and two Nudix proteins (Nudt2 and Nudt16) possessing FAD cap decapping (deFADding) activity. Furthermore, we show an increase of FAD-capped RNA in cells depleted of Nudt16, implicating its role as a deFADding enzyme in cells and report the crystal structure of Nudt16 in the complex with FAD to reveal the structural details of the deFADding activity.

## MATERIALS AND METHODS

### *In vitro* transcription of NAD, FAD and dephosphoCoA-capped RNAs

RNAs containing different cap structures were synthesized by *in vitro* transcription of synthetic double stranded DNA template ϕ2.5-AG-30 containing the T7 ϕ2.5 promoter with an adenosine at the transcription start site (CAGTAATACGACTCACTATT**A**GCCCTCTCTTCCTTCCTTCCTCCTTTCCT). *In vitro* transcription was carried out at 37°C overnight, using HiScribe^TM^ T7 High yield RNA Synthesis kit (New England Biolabs).

To generate 5′ end ^32^P-labeled NAD-capped RNA, ribose ATP was omitted from the reaction and replaced with ^32^P-NAD (PerkinElmer) to initiate transcription. The resulting RNA contains a single ^32^P label within the alpha phosphate of the NAD (Npp*A). Similarly, to generate FAD- or dephosphoCoA-capped RNAs containing a ^32^P-Guanosine at the +2 position, FAD (Sigma) and dephosphoCoA (Sigma) were the only adenosine containing molecules in the mixture to initiate transcription. The reaction was carried out in the presence of [α-^32^P]GTP to incorporate a single ^32^P-label at the +2 position within the capped RNA.

### RNA *in vitro* deNADding, deFADding and deCoAping assays

Recombinant Nudix proteins with an N-terminal 6 × His-tag were purified using His Bind Resin (Novagen) according to the manufacturer's instructions with minor modifications as described (14). The ^32^P-NAD-cap labeled or ^32^P-G (+2)-labeled FAD and dpCoA capped RNAs were incubated with 50 nM of recombinant protein in decapping buffer (10 mM Tris–HCl [pH 7.5], 100 mM KCl, 2 mM dithiothreitol (DTT), 2 mM MgCl_2_ and 2 mM MnCl_2_) as previously described and incubated at 37°C for 30 min (15). Reactions were stopped by heating at 90°C for 2 min. For analysis of deNADding products, reactions were treated with 1 U of Nuclease P1 (Sigma-Aldrich) for 30 min at 37°C. For deFADding and deCoAping product detection, following heat inactivation at 55°C for 5 min, RNAse T1(10U) was directly added to the reaction without further purification. Decapping products were resolved by polyethyleneimine-cellulose thin-layer chromatography (TLC) (Sigma-Aldrich) and developed in 0.45 M (NH_4_)_2_SO_4_ (for deNADding) and in 3M (NH_4_)_2_SO_4_ for deFADding and deCoAping in a TLC chamber at room temperature. TLC plates were exposed onto phosphorimager plates and reaction products were visualized with an Amersham Typhoon Biomolecular Imager (Ge Healthcare).

### Cell culture and generation of CRISPR knockout cell lines

Human embryonic kidney HEK293T cells were obtained from ATCC. Cells were cultured in DMEM medium (Thermo Fisher Scientific) supplemented with 10% fetal bovine serum (Atlanta Biologics) and antibiotics (100 units/ml penicillin and 100 μg/ml of streptomycin) under 5% CO_2_ at 37°C. The three different monoclonal HEK293T cell lines harboring CRISPR-Cas9n double-nick generated distinct homozygous deletions inside the *DXO* or *NUDT12* gene genomic region corresponding to their catalytic site have been reported previously (3,8). Nudt2 and Nudt16 knockout lines were generated using similar CRISPR-Cas9n technology with two Nudt2-gRNAs and two Nudt16-gRNAs designed to target genomic regions of *NUDT2* and *NUDT16*, at sites encoding residues for their respective catalytic sites. The sequences for the guide RNA (gRNA) oligos are provided in Supplementary Table S1. The genomic modification was screened by polymerase chain reaction and confirmed by sequencing and a western blot of the pool of three clones for Nudt2-KO and Nudt16 -KO is shown in Supplementary Figure S2.

### NAD-cap detection and quantitation (NAD-capQ)

HEK293T cells (6 × 10^6^) were seeded in 100 mm plates a day before the experiment and cells were collected for RNA extraction at ∼80% confluency. NAD-capQ was carried out as previously described (15) (Figure 2A). Briefly, 50 μg of total RNA was digested with 2 U of Nuclease P1 (Sigma-Aldrich) in 20 μl of 10 mM Tris (pH 7.0), 20 μM ZnCl_2_ at 37°C for 1 h to release 5′ end NAD. The control samples lacking Nuclease P1 were prepared by incubating 50 μg of RNA treated with the same reaction condition and supplemented with 5% glycerol lacking the enzyme. The NADH standard curve was generated for each experiment in the same buffer condition as above for assays containing nuclease P1. Following digestion with nuclease P1, 30 μl of NAD/NADH Extraction Buffer (NAD/NADH Quantitation Kit, Sigma-Aldrich) was added to each sample. Fifty microliters of each sample was used in a colorimetric assay according to the manufacturer's protocol (NAD/NADH Quantitation Kit, Sigma-Aldrich) as described (15). Values were corrected for background absorbance and concentrations of NAD and NADH were derived from the standard curves.

### FAD-cap detection and quantitation (FAD-capQ)

FAD cap levels were examined in a fractionated population consisting of short RNA <200 nt in size, which we previously demonstrated could be detected by FAD-capQ (16). The short RNA population was isolated from total RNA (500 μg) by LiCl precipitation (2M final concentration). After 30 min incubation at −80°C, samples were centrifuged at 16 000 × *g* at 4°C for 30 min to precipitate RNAs larger than ∼200 nt. The supernatant containing the short RNA fraction was collected and the appropriate amount of commercially provide RLT buffer was added according to the Qiagen protocol. Two volumes of 100% ethanol were subsequently added to the RNA-RLT buffer mixture and samples were applied to RNeasy columns according to the manufacturer (Qiagen).

FAD-capQ was carried out as previously described (16) (Figure 5A). Briefly, 3 μg of short RNA was digested with 250 ng of SpRai1 in 15 μl reaction containing 50 mM Tris (pH 7.9), 100 mM NaCl, 10 mM MgCl_2_ and 1 mM DTT at 37°C for 2 h to release intact FAD. It should be noted that SpRai1 which releases the intact FAD was used instead of Nuclease P1, which is used in NAD-capQ (15), due to the high background in the subsequent fluorescence assay with nuclease P1. The control samples were prepared by incubating 3 μg of RNA under the same reaction conditions with 250 ng of SpRai1 catalytically inactive double-mutant E199A/D201A. FAD standard curves were prepared each time under the same reaction conditions using increasing concentrations of synthetic FAD-capped RNA. After a 2 h incubation at 37°C, 35 μl of FAD Assay Buffer (FAD Colorimetric/Fluorometric Assay Kit, BioVision) was added to each sample. In the second step, 50 μl samples were used in the fluorometric assay to measure OxiRed probe fluorescence according to the manufacture's protocol (FAD Colorimetric/Fluorometric Assay Kit, BioVision). The fluorescence signal was measured using Amersham Typhoon Biomolecular Imager (GE Healthcare) (Ex/Em = 532/570). FAD concentrations were determined relative to the standard curves following subtraction of background values derived from assays containing the catalytically inactive SpRai1 E199A/D201A double-mutant.

### Protein expression and purification for crystallization

All Nudix proteins used in the present study for *in vitro* assays were purified as described previously (17). For the structural analysis of Nudt16, residues 1–184 of human Nudt16 were cloned into a modified pET28a vector, in-frame with an N-terminal 6×His-tagged yeast SMT3 SUMO gene. The protein was over-expressed in *Escherichia coli* BL21(DE3) cells, induced overnight at 17°C with 0.5 mM Isopropyl ß-D-1-thiogalactopyranoside (IPTG) and harvested using centrifugation. The cells were lysed by sonication and clarified by centrifugation, then the lysate was incubated with 1 ml Ni-NTA (Qiagen). Nudt16 was eluted with 250 mM imidazole, and 2 μM yeast UlpI was added and allowed to cleave at room temperature for 30 min. Nudt16 was then removed from the SUMO tag and UlpI using a HiTrap 5 ml Heparin column (GE Lifesciences) at room temperature, using buffers that contained 20 mM HEPES (pH 7.5), 10 mM βME, 1 mM MnCl_2_, and a gradient of NaCl from 50 mM to 2 M. The protein was then further purified with a HiPrep 16/60 Sephacryl S-200 HR column (GE Healthcare) at room temperature using a buffer with 20 mM HEPES (pH 7.5), 250 mM NaCl, 1 mM MnCl_2_ and 10 mM DTT. The protein was concentrated to 25 mg/ml, supplemented with 5% (v/v) glycerol and flash-frozen in liquid nitrogen and stored at −80°C.

### Protein crystallization

Nudt16 was incubated with 5 mM FAD at room temperature for 5 h. Crystals were obtained by the sitting-drop vapor diffusion method at 20°C using this mixture, with the reservoir solution of 0.2 M ammonium chloride and 20% (w/v) PEG 3350 (JCSG Core Suite I, Qiagen). The crystallization solution for free Nudt16 also contained 100 mM MES (pH 5.5). The crystals were picked after 2 weeks and flash-frozen with liquid nitrogen, using 20% (v/v) glycerol as a cryo-protectant.

### Data collection and structure determination

X-ray diffraction datasets were collected using the NE-CAT 24-ID-C beamline at the Advanced Photon Source. The diffraction images were processed with XDS (18), and solved with molecular replacement using Phaser (part of the CCP4 suite) (19), and the PDB entry 3COU (20) as the search model. This solution was then rebuilt using COOT (21) and refined using Refmac5 (22) and PHENIX (23).

## RESULTS

### Screening of mammalian Nudix proteins for deNADding activity *in vitro*

**Figure 1.**
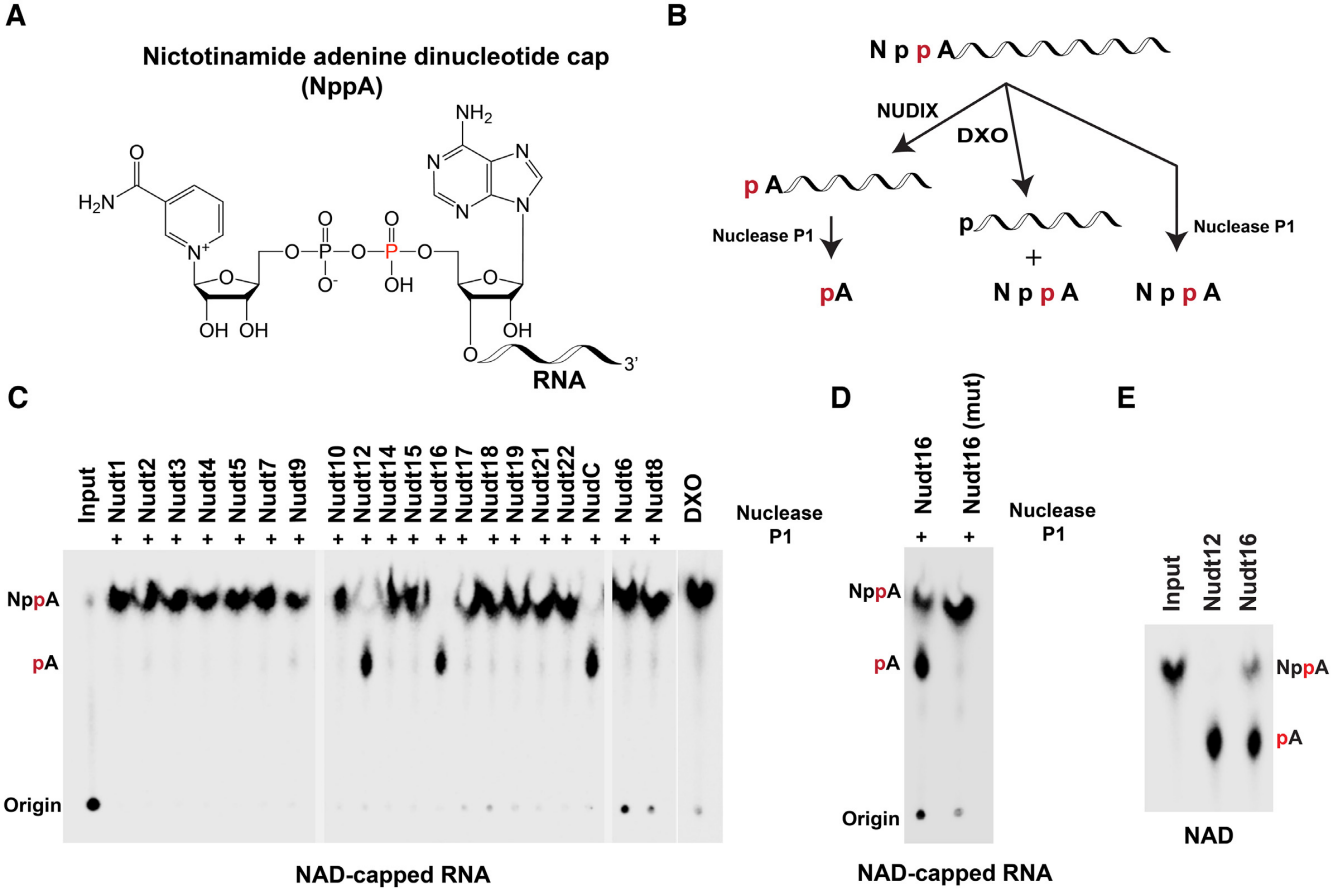
Identification of Nudt16 as a novel deNADding protein *in vitro*. (**A**) Structure of NAD-capped RNA. (**B**) Schematic of the NAD-capped RNA and the expected cleavage products by the indicated enzymes are shown. Nudix proteins cleave within the diphosphate of the NAD while DXO cleaves the phosphodiester linkage after the first adenosine. (**C**) *In vitro* decapping assays were carried out with 50 nM of the indicated recombinant proteins as described in ‘Materials and Methods’ section to screen all mouse Nudix proteins. Bacterial NudC, mouse Nudt12 and DXO proteins were used as positive controls. The ‘+’ indicates lanes treated with Nuclease P1 following Nudix protein reactions. (**D**) Decapping activity of catalytically inactive Nudt16 containing two glutamic acid substitutions to lysine (EE-KK) is shown. (**E**) Nudt16 can also hydrolyze free NAD. Reaction products of ^32^P-labeled free NAD were resolved by TLC developed in 0.45 M (NH_4_)_2_SO_4_. Nudt12 was used as a positive control. The red ‘p’ indicates the position of the ^32^P labeling and markers are denoted on the side of each panel.

Mammalian cells possess 22 Nudix hydrolases (12). We previously showed that seven of them have decapping activity *in vitro* on monomethylated and/or unmethylated capped RNAs (Table 1) and one on NAD-capped RNAs both *in vitro* and in cells (8,17). To assess the deNADding potential of all the Nudix proteins, His-tagged recombinant proteins purified from *E. coli* were incubated with *in vitro* transcribed ^32^P-labeled NAD-capped RNA with ^32^P on the alpha phosphate relative to the RNA (Npp*A-RNA; Figure 1A and B). Unlike DXO that releases intact NAD, Nudix proteins hydrolyze the diphosphate bond (3,8). In the case of the NAD-capped RNA with the ^32^P at the alpha phosphate adjacent to the RNA, the released unlabeled nicotinamide mononucleotide would not be detected by TLC and the labeled p*A-RNA would remain at the origin (Supplementary Figure S1). To enable detection of potential NAD cap hydrolysis, after Nudix protein cleavage, the reaction products were further treated with nuclease to hydrolyze the RNA and release the terminal labeled p*A. Using a TLC based strategy illustrated in Figure 1B, we could detect previously well-characterized products of both DXO and Nudt12 deNADding enzymatic activities– free intact NAD for DXO and p*A for Nudt12 (3,8). The Nudix proteins Dcp2 (Nudt20) and Nudt13 were previously shown to lack deNADding activity (3,8) and not tested. Interestingly, our screening revealed that in addition to Nudt12, Nudt16 also possessed deNADding activity that hydrolyzes the diphosphate bond on NAD-capped RNA *in vitro*. Greater than 99% of the input RNA was reproducibly hydrolyzed by Nudt16 under the assay conditions and protein concentrations used while no activity was detected from the remaining Nudix proteins (Figure 1C and D). Moreover, Nudt16 also hydrolyzed free NAD (Figure 1E), suggesting that similar to Nudt12, Nudt16 is also a Class II deNADding enzyme (8) where it can hydrolyze both the free metabolite as well as the capped derivative.

**Table 1.** Summary of m^7^G and metabolite RNA cap decapping activity of Nudix proteins

	*m^7^Gppp –*	*Gppp –*	*NAD –*	*FAD –*	*dpCoA –*
***Nudt1***					
***Nudt2***	+	+		+	+
***Nudt3***	+	+			
***Nudt4***					
***Nudt5***					
***Nudt6***					
***Nudt7***					+
***Nudt8***					+
***Nudt9***					
***Nudt11***					
***Nudt12***	+	+	+		+
***Nudt13***					
***Nudt14***					
***Nudt15***	+	+			+
***Nudt16***	+	+	+	+	+
***Nudt17***	+	+			
***Nudt18***					
***Nudt19***	+	+			+
***Nudt21***					
***Nudt22***					
***Dcp2***	+				

### Nudt16 is a deNADding enzyme in cells

**Figure 2.**
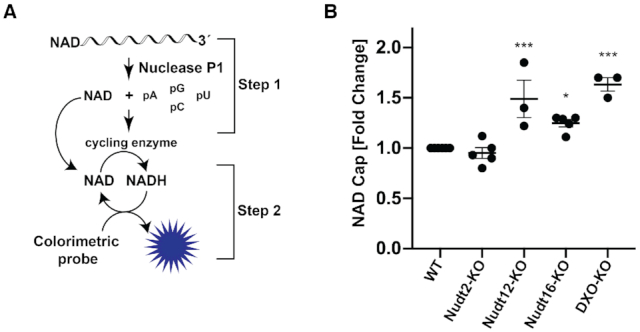
Mammalian Nudt16 is a deNADding enzyme in cells. (**A**) Schematic illustration of NAD-capQ. (**B**) Total RNA from the indicated mammalian cells were subjected to NAD-capQ to detect levels of NAD caps in total cellular RNA. RNA from WT, Nudix protein knockout and DXO knockout cells were used as indicated. Data represent average from at least three independent experiments and are presented in scatter dot plots. Error bars represent ± SEM. Statistical significance level was calculated by one-way ANOVA (*F* = 18.30, df = 2.209, *P* < 0.0001) with a Dunnett's multiple comparison post hoc test, * *P* < 0.05, ** *P* < 0.001, *** *P* < 0.0001.

Having demonstrated Nudt16 can function as a deNADding enzyme *in vitro*, we next determined whether it also modulated NAD capped RNA levels in cells. To assess the effect of Nudt16 deNADding on endogenous NAD-capped RNA, we used the NAD-cap detection and quantitation (NAD-capQ) approach that detects NAD caps en masse (15). NAD-capQ combines the enzymatic properties of nuclease P1 to release intact NAD from the 5′end of NAD-capped RNA with a colorimetric NAD quantitation to detect the released NAD (Figure 2A). Human HEK293T cell lines comprised of a pool of three CRISPR/Cas9n directed *Nudt16* gene knockout monoclonal cell lines (Nudt16-KO), were generated and used (Supplementary Figure S2). Consistent with Nudt16 functioning as a deNADding enzyme in cells, a statistically significant 1.3-fold increase in total NAD-capped RNA was detected by NAD-capQ in cells devoid of Nudt16 compared to the control knockout (herein referred to as wild-type (WT); Figure 2B). RNA from a pool of monoclonal cells containing homozygous disruptions in the *DXO* gene (DXO-KO) or Nudt12 gene (Nudt12-KO), revealed a slightly greater ∼1.5-fold increase in cellular NAD caps (Figure 2B) in agreement with our previous findings (3,8). Moreover, we did not detect significant changes in NAD-capped RNA levels in cells lacking Nudt2 protein, consistent with the lack of detectable deNADding activity for this Nudix protein *in vitro* (Figure 1C). These findings revealed a role for Nudt16 in regulating the levels of NAD-capped RNAs in cells.

### Screening of mammalian Nudix proteins for deCoAping activity *in vitro*

**Figure 3.**
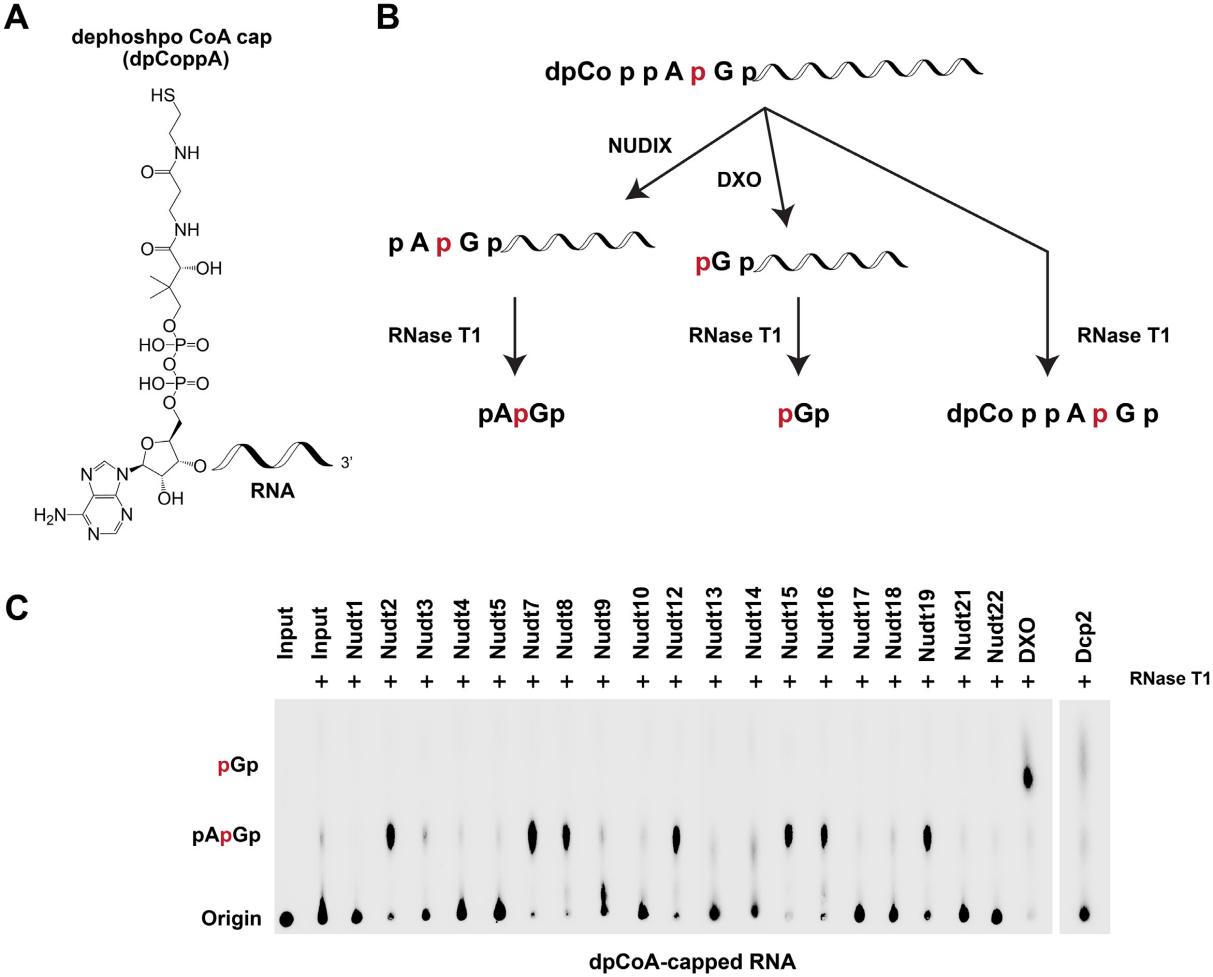
Screen of mouse Nudix proteins for deCoAping activity *in vitro*. (**A**) Structure of dpCoA-capped RNA. (**B**) Schematic of the dpCoA-capped RNA and the expected cleavage products by the indicated enzymes are shown. Nudix proteins cleave within the diphosphate of the dpCoA while DXO cleaves the phosphodiester linkage after the first adenosine. Minimal intrinsic DXO-directed exonuclease activity that would generate p*G results under these assay conditions (16). (**C**) *In vitro* decapping assays with the indicated recombinant mouse Nudix proteins were carried out. Reactions and labeling are as described in the legend to Figure 1.

In addition to NAD, a second nucleotide metabolite, dpCoA, has also been detected on the 5′end of RNAs in at least two bacterial strains, *E. coli* and *Streptomyces venezuelae* by LC-MS/MS (24). Three mammalian Nudix proteins, Nudt7, Nudt8 and Nudt19, have been reported to contain the capacity to hydrolyze free CoA to phosphopantetheine and 3′,5′-ADP (12). Although dpCoA-capped RNAs have not been detected in mammalian cells under standard laboratory growth conditions (11), we nevertheless tested whether any of the Nudix proteins possess the ability to decap dpCoA-capped RNAs *in vitro* (Figure 3A).

To assess the deCoAping activity of Nudix proteins, a modified ^32^P labeling approach was used due to the lack of commercially available ^32^P-labeled dpCoA (see ‘Materials and Methods’ section). dpCoA-capped RNA carrying a single ^32^P at the phosphodiester linkage between the first and second transcribed nucleotides was generated as illustrated in Figure 3B and subjected to decapping by the various Nudix proteins, followed by RNase T1 treatment. Nudix proteins targeting the diphosphate bond within the dpCoA cap would generate pAp*Gp–RNA and subsequently pAp*Gp following RNase T1 digestion (Figure 3B). Conversely, removal of the entire dpCoA from the RNA is expected with the DXO family of proteins, resulting in the formation of p*Gp-RNA and release of p*Gp following RNase T1 treatment (Figure 3B). However, cleavage by RNase T1 in the absence of additional enzymatic activity would generate dpCoAp*Gp that remains at the origin (Figure 3B). As shown in Figure 3C, our screening surprisingly identified seven Nudix proteins—Nudt2, Nudt7, Nudt8, Nudt12, Nudt15, Nudt16 and Nudt19, to possess deCoAping activity *in vitro* significantly above the modest background levels observed with RNase T1 alone (Supplementary Figure S3) under the conditions used. These data reveal an unexpectedly high number of Nudix proteins that are able to cleave dpCoA-capped RNAs *in vitro* in contrast to only two Nudix deNADding proteins.

### Identification of mammalian Nudix proteins possessing deFADding activity

**Figure 4.**
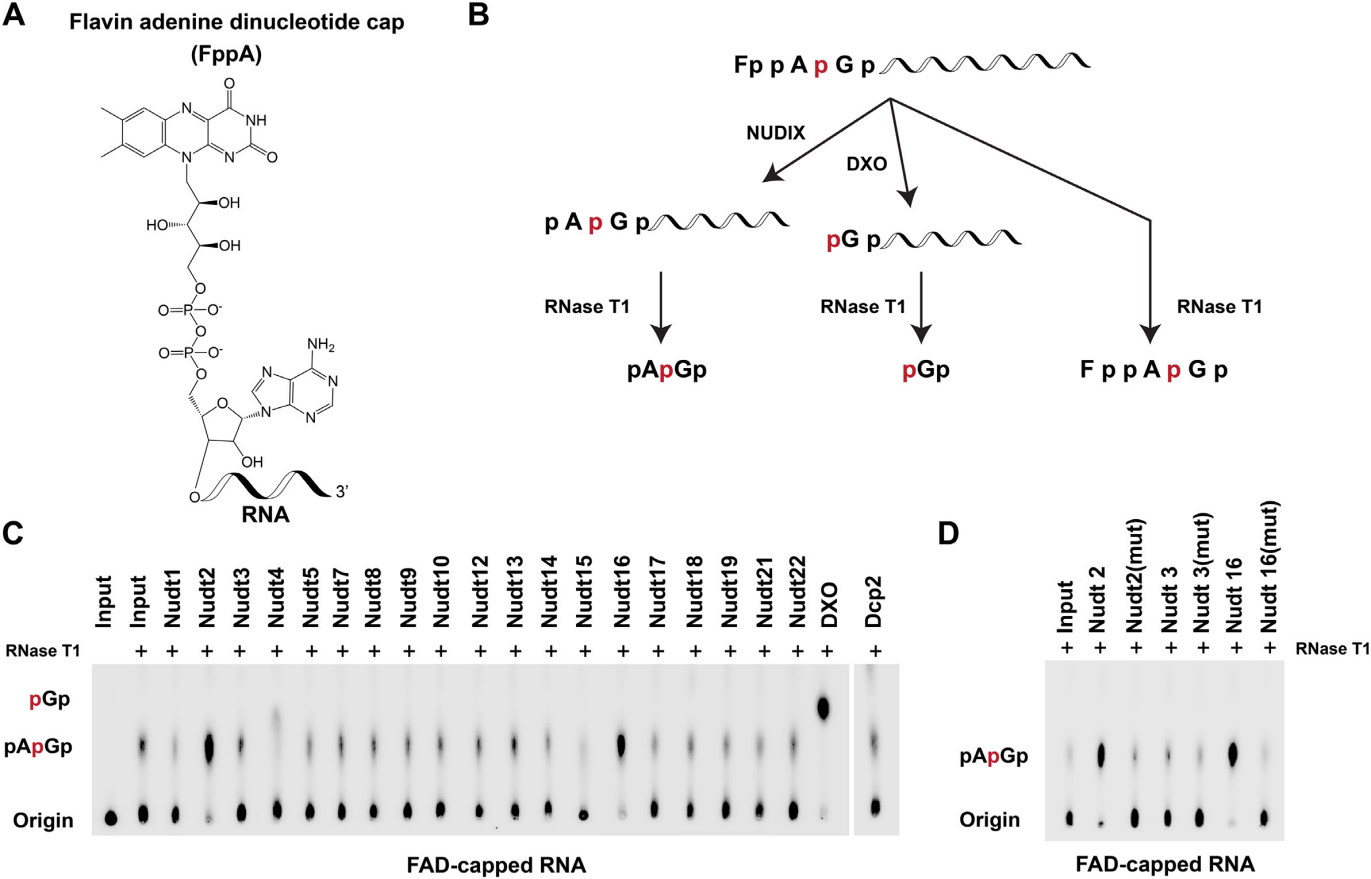
Nudt2 and Nudt16 have deFADding activity *in vitro*. (**A**) Chemical structure of FAD-capped RNA is shown. (**B**) Schematic representation of the likely cleavage site(s) and products of the FAD-capped RNA are shown. Minimal intrinsic DXO-directed p*G exonuclease activity is evident under the assay conditions used (16). (**C**) *In vitro* decapping assays of FAD-capped RNAs by the indicated recombinant mouse Nudix proteins. (**D**) Decapping activity of catalytically inactive Nudt2, Nudt3 and Nudt16 are shown. Highly conserved glutamic acid residues essential for catalysis within the Nudix motif were substituted with lysine in Nudt16 (EE-KK), glutamines in Nudt2 and Nudt3 (EE-QQ). Reactions and labeling are as described in the legend to Figure 1.

FAD-capped RNAs were recently reported in mammalian cells, albeit at low abundance (11) and the DXO family of proteins were identified as robust deFADding enzymes *in vitro* and in cells (16). The presence of multiple deNADding and deCoAping Nudix proteins prompted us to determine whether Nudix proteins can also hydrolyze FAD-capped RNAs. FAD-capped RNAs with a single ^32^P moiety were generated analogous to dpCoA-capped RNAs described above. The panel of Nudix proteins was next evaluated for their propensity to deFAD. DXO cleavage of the FAD-capped RNA removes the intact FAD and generates a labeled 5′ monophosphate RNA that begets p*Gp following RNase T1 exposure (Figures 4B). Hydrolysis by Nudix proteins within the diphosphate linkage of FAD followed by RNase T1 digestion would release pAp*Gp dinucleotide with the central phosphate ^32^P labeled (Figures 4B).

*In vitro* transcribed FAD-capped RNA was incubated separately with each Nudix protein, followed by RNAse T1 digestion. Despite the presence of background pAp*Gp levels generated by RNase T1 alone, Nudt2 and Nudt16 demonstrated deFADding activity (Figures 4C and D) at levels significantly above background (Supplementary Figure S4). Surprisingly, Nudt12, which has previously been shown to hydrolyze free FAD, did not show detectable deFADding activity against FAD capped RNAs. Collectively, our data demonstrate at least two Nudix proteins, Nudt2 and Nudt16 have deFADding activity *in vitro* and may regulate FAD-capped RNAs in cells.

### Nudt16 is a deFADding enzyme in cells

**Figure 5.**
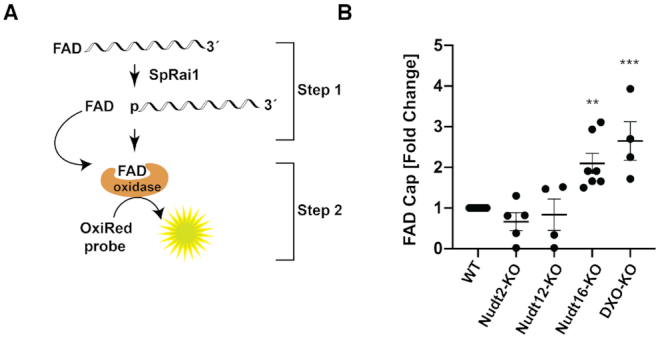
Mammalian Nudt16 is a deFADding enzyme in cells. (**A**) Schematic illustration of FAD-capQ is shown. (**B**) Short RNAs derived from the indicated cells as in Figure 2B were subjected to FAD-capQ to quantitate levels of FAD-capped RNAs. Data represent the average from at least four independent experiments and are presented in scatter dot plots. Error bars represent ± SEM. Statistical significance level was calculated by one-way ANOVA (*F* = 11.98, df = 4.407, *P* < 0.0001) with a Dunnett's multiple comparison post hoc test; ** *P* < 0.001, *** *P* < 0.0001.

To test whether Nudt2 and Nudt16 function as deFADding enzymes in cells, we asked whether any changes in FAD capping can be detected in cells depleted of these enzymes by applying a FAD-capQ assay. FAD-capQ is a two-step enzymatic procedure that determines FAD cap levels in cells by removal of the intact 5′-FAD from RNA by SpRai1 cleavage of the phosphodiester bond between the first and second encoded nucleotides within the RNA cap, followed by quantitation of the released 5′-FAD by an FAD fluorometric assay (16) (Figure 5A). We recently reported the presence of FAD caps on short RNAs in human HEK29T cells by this method (16) and therefore assessed the level of FAD-caps on short RNAs in cells lacking Nudt2 or Nudt16 enzymes. A LiCl precipitation approach coupled with silica-based RNA purification was used to obtain short RNAs less than ∼200 nt in length. Equal amounts of short RNA fractions isolated from WT, Nudt2-KO, Nudt12-KO, Nudt16-KO and DXO-KO cells were subjected to FAD-capQ. RNAs from Nudt12-KO were used as a negative control for the assay since Nudt12 does not possess enzymatic activity on FAD-capped RNA *in vitro* (Figure 4C), whereas previously reported increase of FAD-capped RNAs in DXO-KO cells (16) served as a positive control. Interestingly, FAD-capQ analysis showed a significant ∼2-fold increase of FAD-capped RNAs in cells lacking Nudt16, but not in cells lacking Nudt2 (Figure 5B). The observed increase of FAD-capped RNAs in the Nudt16-KO cells was comparable to that of FAD-capped RNAs in DXO-KO cells (16) (Figure 5B). This result supports a functional role for Nudt16 in regulating the fate of FAD-capped short RNAs in mammalian cells, while a similar function cannot be attributed to Nudt2 at least under the growth conditions and the cells employed.

### Structural basis for recognition of FAD by human Nudt16

To understand the molecular mechanism for the recognition of FAD-capped RNA by Nudt16, we determined the crystal structure of human Nudt16 in complex with FAD at 2.7 Å resolution and the structure of free human Nudt16 at 2.3 Å resolution (Table 2). Both structures reported here are in new crystal forms compared to those in the Protein Data Bank (20,25–26), and the free Nudt16 crystal contains two dimers in each asymmetric unit. The overall structure of Nudt16 is similar to those reported earlier, with rms distances of approximately 0.6 Å for the dimer. The loop containing residues 101–106, located near the ribose of FAD in the complex, shows substantial variations among the structures and has weak electron density in the free Nudt16 structure. The equivalent loop (L8 loop) in Nudt16-like protein (also known as syndesmos and TIRR) is involved in interactions with 53BP1 Tudor domain (27,28).

**Table 2.** Summary of crystallographic information

Structure	Human Nudt16 + FAD	Free human Nudt16
**Data Collection**		
Space group	*P*3_2_	*P*2_1_2_1_2_1_
Cell dimensions		
*a*, *b*, *c* (Å)	64.7, 64.7, 77.8	85.7, 137.9, 55.0
α, β, γ (°)	90, 90, 120	90, 90, 90
Resolution (Å)^a^	45.5–2.7 (2.87–2.7)	46.3–2.3 (2.44–2.3)
*R*_merge_ (%)	9.6 (70.5)	6.6 (76.5)
CC_1/2_	0.996 (0.602)	0.998 (0.746)
I/σI	11.4 (1.6)	10.5 (1.4)
Completeness (%)	99.5 (99.7)	97.6 (95.3)
No. of reflections	9929	28854
Redundancy	4.3 (4.4)	4.1 (4.2)
**Refinement**		
Resolution (Å)	45.5–2.7 (2.77–2.7)	46.3–2.3 (2.39–2.3)
*R*_work_ (%)	15.9 (38.9)	22.4 (37.6)
*R*_free_ (%)	23.2 (47.6)	28.7 (46.3)
Number of atoms	2641	5088
Protein	2524	5030
Ligand/Ion	80	2
Water	37	56
B-factors (Å^2^)	61.1	63.9
Protein	61.0	64.0
Ligand	69.7	55.2
Water	48.8	54.4
r.m.s.d.		
Bond lengths (Å)	0.007	0.010
Bond angles (°)	1.5	1.2
Ramachandran plot		
Favored (%)	88.4	93.6
Allowed (%)	11.0	6.1
Outliers (%)	0.6	0.3

^a^The numbers in parentheses are for the highest resolution shell.

**Figure 6.**
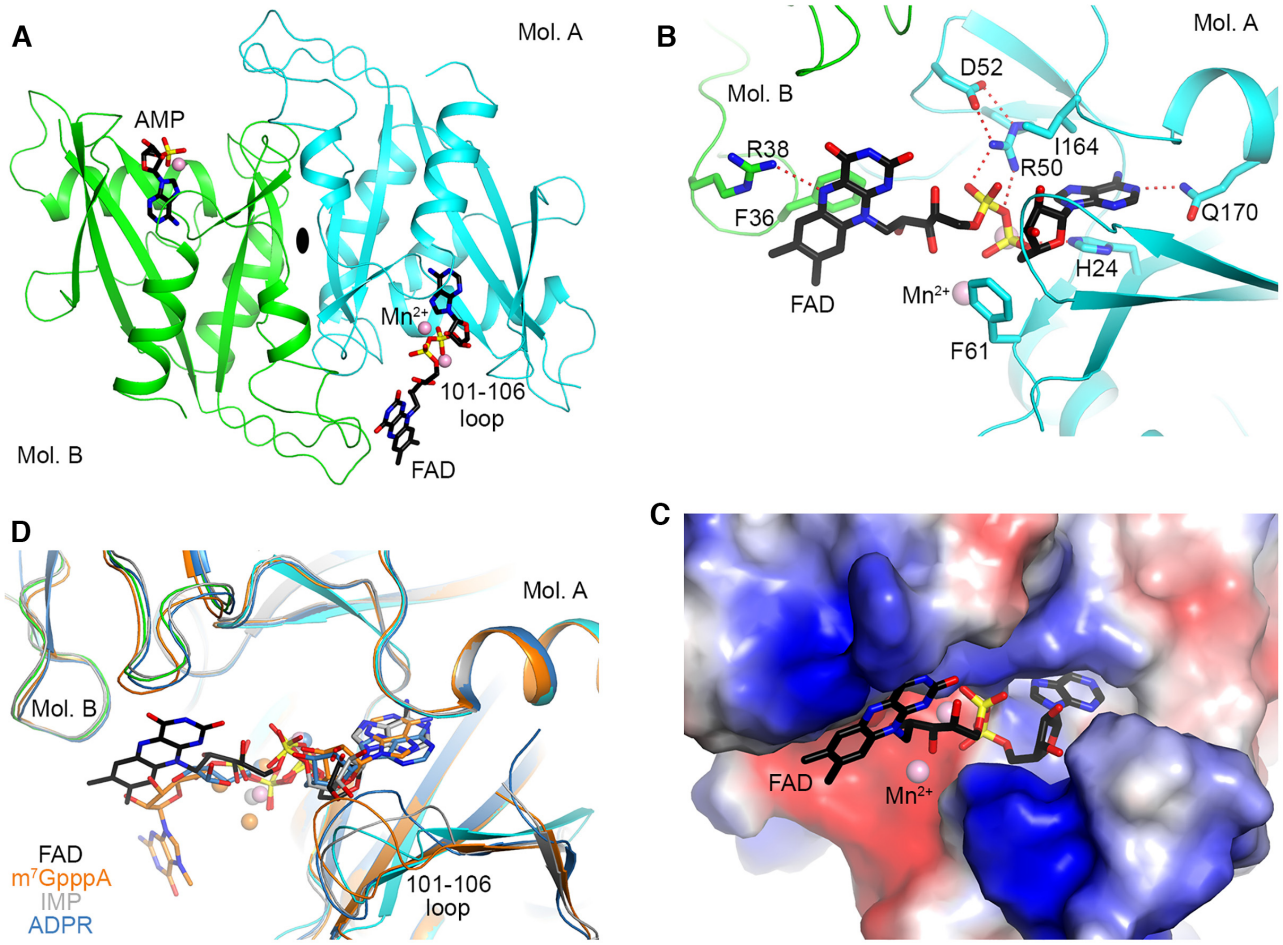
Crystal structure of human Nudt16 in complex with FAD. (**A**) Schematic drawing of the crystal structure of human Nudt16 in complex with FAD. The two monomers of Nudt16 are colored in cyan and green, respectively. FAD is observed in the active site of one monomer, while AMP is observed in the other (both shown as stick models with carbon atoms in black). The Mn^2+^ ions are shown as spheres in pink. (**B**) Detailed interactions between FAD and Nudt16. Hydrogen-bonding interactions are indicated with the dashed lines in red. (**C**) Electrostatic surface of the active site region of Nudt16. The AMP portion is mostly buried in the active site, while the flavin ring and ribitol group are mostly exposed. (**D**) Overlay of the structures of Nudt16 in complex with FAD, m^7^GpppA cap, IMP and ADPR. Large conformational differences are also seen for the 101–106 loop. The figure was produced with PyMOL (www.pymol.org).

FAD is bound in the active site of Nudt16 (Figure 6A). An entire FAD molecule is observed in the active site of one monomer, while only AMP is observed in the active site of the other monomer (Figure 6A), likely due to hydrolysis during crystallization and consistent with the deFADding activity of Nudt16. In this binding mode, the 3′ hydroxyl group of the ribose is exposed to the solvent, and the first few nucleotides of the RNA body could have interactions with the loop containing residues 101–106.

The adenine base of FAD is π-stacked with the side chain of His24 on one face and positioned against Ile164 on the other (Figure 6B). The N1 atom of adenine is hydrogen-bonded to the side chain of Gln170, and the N6 atom is located near the main-chain amide nitrogen of Phe57. The α phosphate is coordinated to a Mn^2+^ ion in the active site and has hydrogen-bonding interactions with His24. The β phosphate is involved in ionic interactions with the side chain of Arg50. The ribitol group of FAD is placed near the side chain of Phe61 but is mostly exposed to the solvent. The flavin ring is π-stacked against the side chain of Phe36, from the other monomer of the dimer, while the other face of the flavin ring is exposed to solvent (Figure 6C). The side chain of Arg38, also from the other monomer, has hydrogen-bonding interaction with the N5 atom of the flavin ring. The flavin ring also has interactions with another Nudt16 molecule in the crystal, and therefore its conformation might also be stabilized by crystal packing.

The AMP portion of FAD is bound at a location generally similar to that of equivalent moiety in other compounds, such as the m^7^GpppA cap (25), inosine monophosphate (IMP) (20) and ADP-ribose (26) (Figure 6D), suggesting that the deFADding activity shares the same catalytic mechanism as these other reactions. In comparison, the position of the flavin ring is quite different from that of the m^7^G base. In fact, the m^7^G base does not appear to have any direct contact with the protein.

## DISCUSSION

Overwhelming evidence now supports the existence of nucleotide metabolite caps on the 5′end of RNAs. NAD-capped RNAs have been detected in bacteria, yeast, plants and mammals (7); dpCoA caps in bacteria and FAD caps in bacteria, yeast and mammals (11,16). Collectively, these findings have uncovered a previously unknown mechanism for regulatory gene expression through nucleotide metabolite-directed control of RNA fate. Furthermore, at least two proteins, DXO and Nudt12, have been identified to cleave NAD caps in mammalian cells (3,8), each targeting distinct subsets of NAD-capped RNAs, suggesting additional deNADding proteins likely exist in mammalian cells. In the present study we evaluated all mammalian Nudix proteins for potential deNADding, deCoAping and deFADding enzymatic activities *in vitro*. In addition to DXO and Nudt12, we demonstrated Nudt16 also possesses deNADding activity, while seven Nudix proteins (Nudt2, Nudt7, Nudt8, Nudt12, Nudt15 Nudt16 and Nudt19) can remove a dpCoA cap from RNAs, and two Nudix proteins (Nudt2 and Nudt16) contain deFADding activity. These findings demonstrate a network of Nudix proteins have the capacity to hydrolyze metabolite capped RNAs in mammalian cells and identifies previously unknown proteins that may function in the coordination of cellular metabolism to RNA metabolism.

Identification of NAD-capped RNAs from Nudt12-KO cells revealed Nudt12 selectively targets a subset of NAD capped RNAs including RNAs involved in cellular metabolism (8). Similarly, a subset of small nucleolar (sno)RNAs, in addition to mRNAs, that contain a 5′end NAD cap are specifically deNADded by the DXO protein (3). At present, the specific NAD-capped RNA substrates for Nudt16 are not known. It is tempting to speculate that Nudt16 may also regulate the deNADding of select NAD-capped RNAs involved in specific cellular pathways. In particular, Nudt16 was initially characterized as an RNA decapping protein in *Xenopus laevis* with high affinity binding to U8 snoRNAs (29,30). Whether high affinity binding to an mRNA would equate to preferential deNADding is unclear although in the case of the human Dcp2 decapping enzyme, enhanced binding led to more efficient decapping *in vitro* (31,32). Selective targeting of specific NAD-capped RNAs could account for the modest effect of Nudt16 on total NAD cap levels. Alternatively, the presence of at least three different deNADding enzymes in mammalian cells may indicated redundancy and require a background with multiple knockouts for optimal Nudt16 deNADding activity detection. Nevertheless, NAD-capQ analysis of total NAD-capped RNAs in Nudt16-KO cells validates a cellular role for Nudt16 as a deNADding enzyme.

The presence of dpCoA-capped RNAs in bacteria prompted us to test whether mammalian Nudix proteins can hydrolyze dpCoA-capped RNAs. An unexpectedly large number of proteins were found to remove the dpCoA cap from RNA. We identified seven Nudix proteins along with DXO that possess deCoAping activity (16). Three of the deCoAping competent Nudix proteins—Nudt7, Nudt8 and Nudt19 can also hydrolyze free CoA, CoA esters, dpCoA and their derivatives (12). These three enzymes are analogous to the Nudt12 and Nudt16 class 2 deNADding enzymes that can hydrolyze both the free NAD nucleotide metabolite and NAD-capped RNAs (8). The remaining four deCoAping Nudix proteins (Nudt2, Nudt12, Nudt15 and Nudt16) have not previously been implicated in CoA biology. It is unclear why cells would possess so many deCoAping enzymes, but the presence of multiple deCoAping enzymes (at least *in vitro*) could be one explanation for the lack of detectable dpCoA-capped RNAs in mammalian cells (11). It is plausible that all or a subset of the seven Nudix proteins and DXO possess robust deCoAping activity in cells as well. Future studies with multiple knockouts of the genes encoding these proteins along with more sensitive detection methodologies would address whether dpCoA-capped RNAs exist in mammalian cells.

Our analysis of enzymes hydrolyzing FAD caps *in vitro* uncovered two Nudix proteins, increasing the number of proteins capable of deFADding to three enzymes, Nudt2 and Nudt16 (Figure 4) and DXO (16). Similar to DXO, Nudt16 can modulate levels of short FAD-capped RNAs in cells as assessed by FAD-capQ analysis of cells disrupted for the gene encoding Nudt16. In contrast, we were unable to detect differences in levels of FAD-capped RNAs between WT cells and cells lacking Nudt2 protein suggesting Nudt2 may not function on FAD-capped RNAs in cells. However, it is difficult to interpret this negative result since Nudt2 may only modulate a small subset of RNAs under normal growth conditions, which would not be evident by analyzing total FAD-capped RNAs. Methodologies that can selectively isolate FAD-capped RNAs will be needed to address this possibility. Collectively, our data demonstrate that Nudt16 and DXO function as deFADding enzymes and likely modulate levels of FAD-capped RNAs in cells. The function(s) of an FAD cap in cells remains to be determined.

A comprehensive list of currently known biochemical activities of the mammalian Nudix proteins on capped RNA is presented in Table 1. Among the Nudix proteins, Nudt16 is the most promiscuous. Similar to DXO, it can hydrolyze RNAs harboring an m^7^G cap (17), unmethylated cap (17), NAD cap (Figure 1), dpCoA cap (Figure 3) and FAD cap (Figure 4) on the 5′end. The pleiotropic nature of Nudt16 is further evidenced by its hydrolysis of (deoxy)inosine diphosphatase in the nucleus to protect cells from potential adverse effects of (d)ITP incorporation into DNA (33). The crystal structures of Nudt16 in complex with m^7^GpppA (25), inosine (20) and FAD (Figure 6D) are consistent with its pleotropic activity on multiple cap substrates, as the m^7^G and flavin moieties are at the surface with minimal protein contacts.

Beside Nudt16, Nudt2 and Nudt12 also have a broad range of capped substrates that they can decap. Both enzymes hydrolyze unmethylated Gppp- (17), m^7^Gppp- (17) and dpCoA-capped RNAs (Figure 3) yet interestingly differ in their propensity to decap NAD- and FAD-capped RNAs. Whereas Nudt2 possesses deFADding activity on FAD-capped RNAs *in vitro* (Figure 4), it does not hydrolyze NAD-capped RNA (Figure 1). Conversely, Nudt12 is capable of deNADding (Figure 1), but unable to deFAD (Figure 4). The deNADding activity of Nudt12 preferentially targets RNAs involved in cellular metabolism (8), the functional significance of Nudt2 deFADding is still unclear. We were unable to detect alterations in FAD-capped RNA levels in the short RNA fraction in the absence of Nudt2. One important caveat remains, however. Our detection of FAD-capped RNAs by FAD-capQ is limited to small RNAs and to the standard growth conditions employed (16). Potential effect of Nudt2 on specific longer RNAs or function under stress conditions in cells may still be possible and remains to be addressed.

Surprisingly, the three proteins that are capable of deNADding and deFADding are among the list of eight Nudix proteins (Nudt2, 3, 12, 15, 16, 17, 19 and 20 [Dcp2]) previously demonstrated to possess mRNA decapping activity (17). Interestingly, Nudt7 and Nudt8, which were not identified with canonical decapping activity (17), nevertheless do harbor decapping activity with a restricted substrate specificity of dpCoA-capped RNA. This raises the question of whether any of the remaining Nudix proteins without previously demonstrated decapping activity may function on other as yet uncharacterized nucleotide metabolite RNA caps. Our studies reveal the diverse activities of the Nudix family of proteins on an array of nucleotide metabolite caps and establish a foundation for future functional analyses of these novel RNA caps, their decapping proteins, and their interface to cellular metabolism.

## Supplementary Material

gkaa402_Supplemental_File
