# Supplementary material for: Mammalian Nudix proteins cleave nucleotide metabolite caps on RNAs
Source: Nucleic Acids Res. 2020 May 20;48(12):6788–98. doi: 10.1093/nar/gkaa402 (PMC7337524; doi:10.1093/nar/gkaa402)
Supplement: gkaa402_Supplemental_File [file gkaa402_supplemental_file.pdf]

Supplemental

Mammalian Nudix proteins cleave nucleotide metabolite caps on RNAs

Sunny Sharma,[1] Ewa Grudzien-Nogalska,[1*] Keith Hamilton,[2*]

Xinfu Jiao,[1] Jun Yang,[1] Liang Tong,[2] and Megerditch Kiledjian[1#]

[1]Department of Cell Biology and Neuroscience

Rutgers University

Piscataway, NJ 08854, USA

[2]Department Biological Sciences

Columbia University

New York, NY 10027

* -equal contribution

[#] -corresponding author email: kiledjian@biology.rutgers.edu

Supplementary Figures

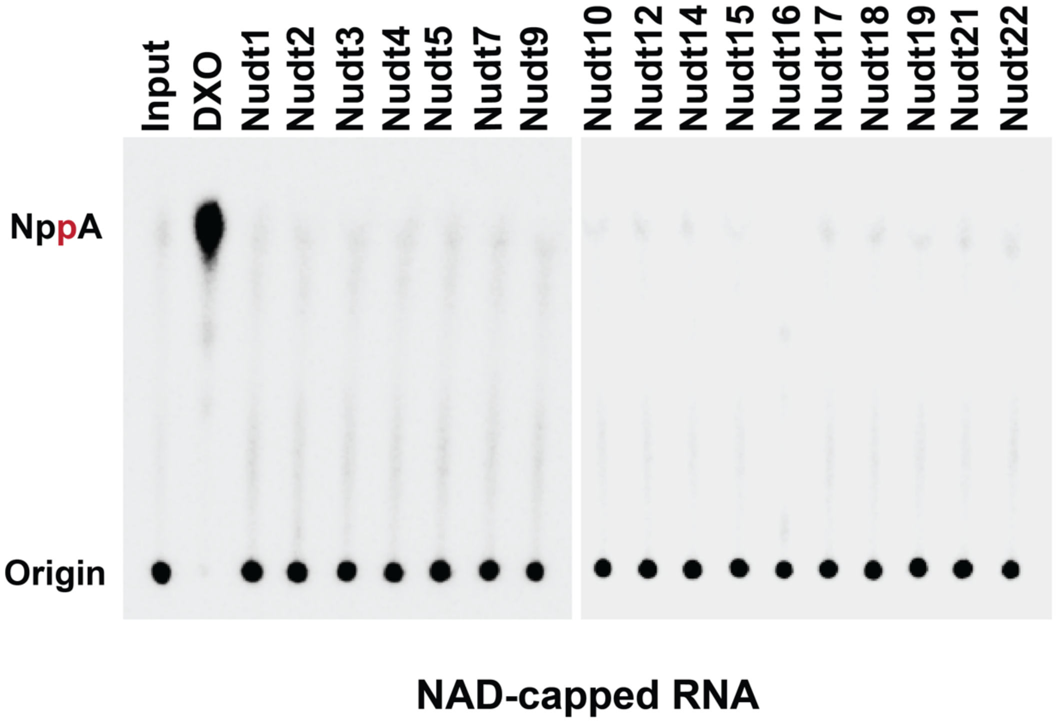

Supplementary Figure 1. deNADding activity without the use of nuclease P1. DXO releases intact NAD that can be detected using TLC. However, deNADding products by Nudix proteins which would remove nicotinamide monophosphate from the NAD cap and leave the ^{32}P on the RNA at the origin. Reactions require subsequent nuclease P1 treatment to enable deNADding detection (please see Figure 1B and 1C).

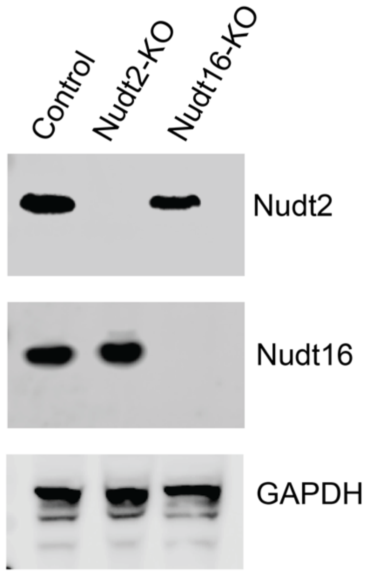

Supplementary Figure 2 Western blot analysis of Nudt2 and Nudt16 protein levels in HEK293T Control, Nudt2-KO and Nudt16-KO cell lines. Glyceraldehyde 3-phosphate dehydrogenase (GAPDH) was used as a loading control.

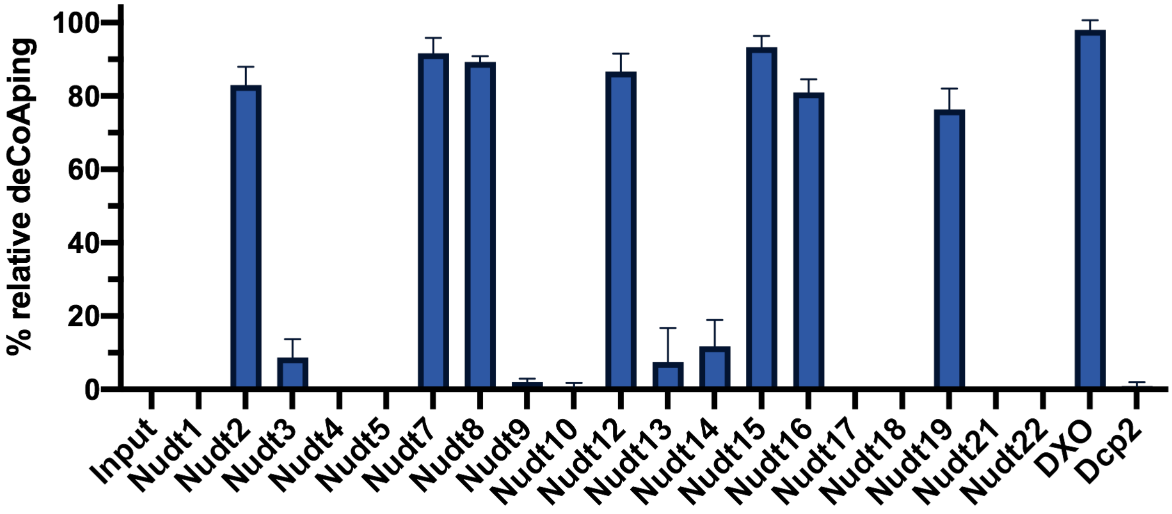

Supplementary Figure 3. **Quantitation of Nudix protein deCoAping activity.** deCoAping activity for each Nudix protein from Figure 3C quantitated by ImageJ are plotted. Values are presented relative to the background of RNase T1 treated input RNA in the absence of Nudix protein. Data were derived from three independent experiments with error bars representing +/- SD.

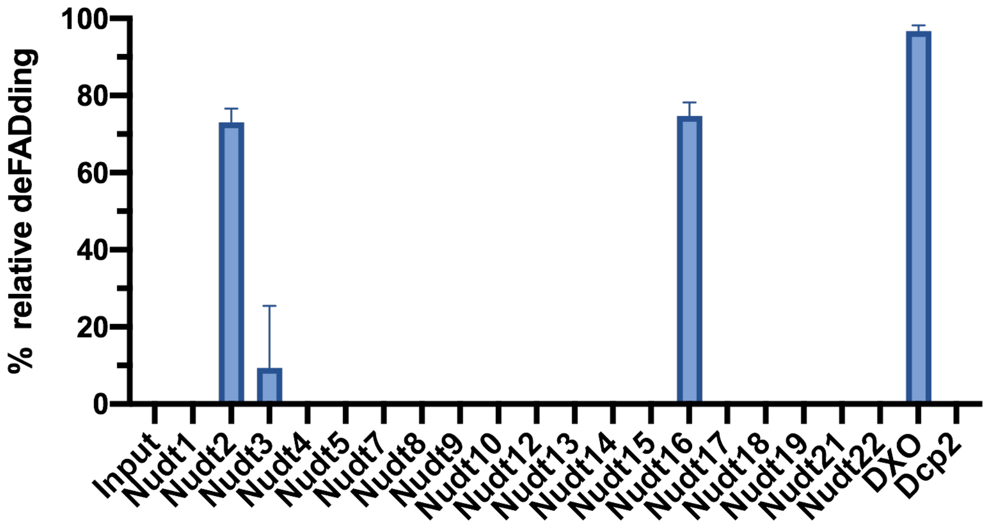

Supplementary Figure 4. Quantitation of Nudix protein deFADding activity. deFADding activity for each Nudix protein from Figure 4C quantitated with ImageJ are plotted. Normalized values relative to the background of RNase T1 treated input RNA in the absence of Nudix protein are presented. Data were derived from three independent experiments with error bars denoting +/- SD.

Supplementary Table S1

Item	Sequence	Comments
**Nudt2-gRNA-1**	AGGCCAGCUGACCAUUAUUG	Used for Nudt2 KO
**Nudt2-gRNA-2**	UUCCUCUUGGGUCUCCCUCA	Used for Nudt2 KO
**Nudt16-gRNA-1**	AGCGGCUGCCGCUUUCCGCG	Used for Nudt16 KO
**Nudt16-gRNA-2**	CGCGGUUCAGCCCGUCCUCU	Used for Nudt16 KO
**Nudt2 GP1**	GGCACATGCCTGTTGTTCTG	Used in combination with GP2 and GP3 for qPCR and sequencing
**Nudt2 GP2**	AGAGGAAGCAGGCATAGAAGC	Used in combination with GP1 for qPCR
**Nudt2 GP3**	GGTAGGCTTGGTGCTCATGG	Used in combination with GP for sequencing
**Nudt16 GP1**	CGCCATACTGGTGAGAAGGG	Used in combination with GP2 and GP3 for qPCR and sequencing
**Nudt16 GP2**	CGCAGCTCGCGGTTCAG	Used in combination with GP1 for qPCR
**Nudt16 GP3**	AAAGACTTTTCTCCAGGGATGC	Used in combination with GP for sequencing
